# Patient Perspectives of Dignity, Autonomy and Control at the End of Life: Systematic Review and Meta-Ethnography

**DOI:** 10.1371/journal.pone.0151435

**Published:** 2016-03-24

**Authors:** Andrea Rodríguez-Prat, Cristina Monforte-Royo, Josep Porta-Sales, Xavier Escribano, Albert Balaguer

**Affiliations:** 1 Faculty of Humanities, Universitat Internacional de Catalunya. Barcelona. Spain; 2 Nursing Department, School of Medicine and Health Sciences, Universitat Internacional de Catalunya, Barcelona, Spain; 3 Palliative Care Service, Institut Català d’Oncologia, Barcelona, Spain; 4 School of Medicine and Health Sciences, Universitat Internacional de Catalunya, Barcelona, Spain; S.G.Battista Hospital, ITALY

## Abstract

**Background:**

Research in the end-of-life context has explored the sense of dignity experienced by patients with advanced disease, examining the factors associated with it. Whereas certain perspectives regard dignity as an intrinsic quality, independent of external factors, in the clinical setting it is generally equated with the person’s sense of autonomy and control, and it appears to be related to patients’ quality of life. This study aims to explore the relationship between perceived dignity, autonomy and sense of control in patients at the end of life.

**Methods:**

We conducted a systematic review and meta-ethnography using reciprocal translation and line-of-argument synthesis. The search strategy used MeSH terms in combination with free-text searching of the Pubmed, Web of Science, CINAHL, PsycINFO and Cochrane databases, from their inception until 2015. This identified 186 articles, after excluding duplicates. The inclusion criterion was primary qualitative studies in which dignity, autonomy and control at the end of life were explored. Studies were evaluated using the CASP guidelines.

**Results:**

Twenty-one studies recording the experiences of 400 participants were identified. Three themes emerged: a) dignity mediated by the loss of functionality, linked to the loss of control; b) dignity as identity; and c) autonomy as a determining factor of perceived dignity, understood as the desire for control over the dying process and the desire for self-determination. We propose an explanatory model which highlights that those patients with an intrinsic sense of dignity maintained a positive view of themselves in the face of their illness.

**Conclusion:**

This synthesis illustrates how dignity and autonomy are intertwined and can be perceived as a multidimensional concept, one that is close to the notion of personal identity. The ability to regard dignity as an intrinsic quality has a positive impact on patients, and the design of care strategies should take this into account.

## Introduction

Safeguarding the dignity of patients at the end of life (EOL) has become a key objective of clinical practice [1–4]. Numerous studies have sought to clarify what is meant by dignity [4–11], to identify the variables associated with it [12–15], to examine how it is perceived by patients, families, and professionals [2,15–19], and to explore ways of assessing and enhancing it [20–22].

Broadly speaking, there are two ways in which the notion of dignity is evoked [6,7,23]. One is to consider it as something intrinsic and ontological, what some authors refer to as *basic dignity*. From this point of view, dignity is an irrevocable feature of personhood that does not depend on, or vary, according to circumstances. The second perspective refers to what is called *dynamic dignity*, that is, a personal quality that is related to people’s perception of themselves and of the context in which they live. In the present study, dignity is considered to be a fundamentally intrinsic feature of the human individual [11], although it is acknowledged that what it entails in practice will depend on how patients see themselves and are seen by others, and also on how the nature of the illness in question affects the person’s life and identity.

In the EOL context, another key issue is how the perception of dignity is mediated by the person’s sense of autonomy or control. Although the two terms (autonomy and dignity) are sometimes regarded as distinct concepts, this is not always the case in the EOL setting. Indeed, a loss of autonomy or control among patients is often interpreted as a loss of self, and of the sense of dignity [24].

Research conducted to date on the perception of dignity and autonomy has contributed to an understanding of the needs and concerns of patients facing the EOL, and of the kind of care they require in order to improve their wellbeing. However, although the terms autonomy and dignity are frequently used in the literature [25] the link between them remains ambiguous. Paradoxically, dignity—especially when it is understood as autonomy—often appears as a key argument in clinical, legal, and philosophical debates, where it may be invoked to support opposing positions. A clear example of this is how the notion of dignity may be used both to support and challenge the act of euthanasia and assisted suicide, with opposing conclusions being reached on the basis of the same principles [23].

Given the lack of clarity and consensus that has been highlighted by many authors [2,4,5,9,23] the aim of this study was to explore, by means of a systematic review and interpretative synthesis, the primary qualitative studies that have focused on autonomy and control as mediators of the patient’s dignity at the EOL, as perceived by patients, families and health professionals. The goal in doing so was to analyse how the relationship between autonomy and dignity is interpreted in this context.

## Methods

The search strategy combined MeSH terms with free-text searching and was applied to Pubmed, Web of Science, CINAHL, PsycINFO and the Cochrane Library, from their start date until November 2015. Several trials were required to achieve a sensitive and specific search strategy (see Table 1). The reference lists of the retrieved studies were also reviewed.

**Table 1:** Final search terms for the strategy applied in the databases.

Patient	1	Patient [MeSH]
	2	Disease [MeSH]
	3	Illness [MeSH]
	4	1 or 2 or 3
End of life	5	Death [MeSH]
	6	Palliative [Text Word]
	7	End of life [Text Word]
	8	Hospice [MeSH]
	9	5 or 6 or 7 or 8
Die with dignity	10	Dignity [Text Word]
	11	Dignified dying [Text Word]
	12	Dignified death [Text Word]
	13	Die with dignity [Text Word]
	14	10 or 11 or 12 or 13
Autonomy	15	Control [MeSH]
	16	Autonomy [MeSH]
	17	Self-determination [MeSH]
	18	15 or 16 or 17
Final strategy	19	4 and 9 and 14 and 18

**Fig 1:**
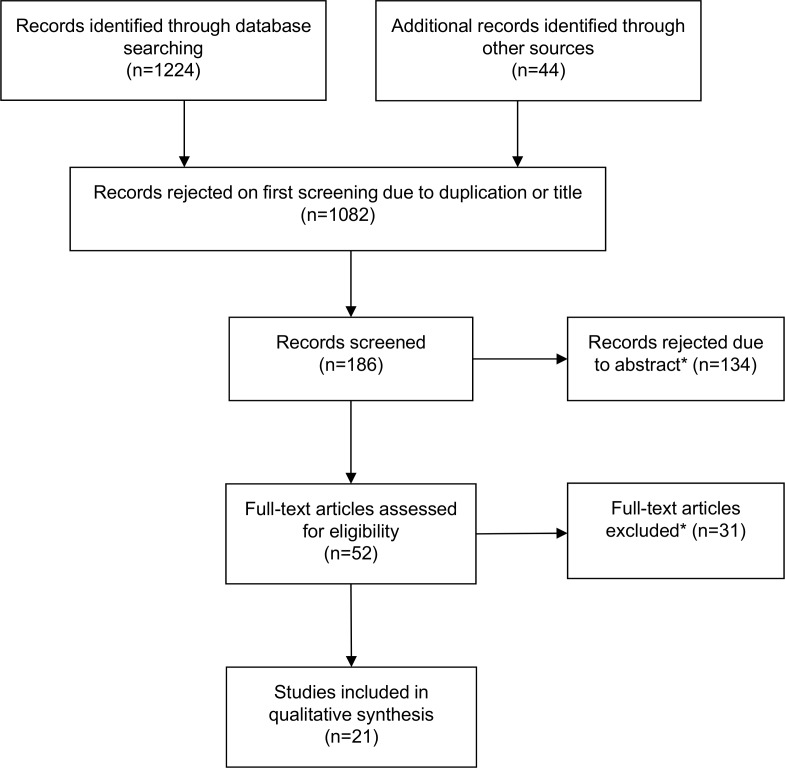
Flowchart of search results. *Studies excluded because of method, participants or topic.

The inclusion criteria were as follows: primary qualitative studies in which dignity, autonomy and control at the end of life were explored (patients with an advanced disease and older people), as perceived by patients themselves, by their relatives and/or by health professionals. Studies involving paediatric samples were excluded. A total of 21 studies were included in the systematic review. Fig 1 shows the process of study selection.

A systematic review and interpretative synthesis was performed using the meta-ethnography approach developed by Noblit and Hare [26], This is an inductive method that involves making constant comparisons of the concepts found in different qualitative studies in order to enable a critical examination of a phenomenon and to extract common conclusions [26].

This study did not require ethical approval. Each of the included studies had been approved by the corresponding local ethics committee.

### Quality Assessment

The studies included were evaluated using the CASP guidelines for qualitative research (see S1 Table) [27], with the exception of three reports [28–30], whose design was not compatible with these guidelines. However, we considered that all the studies could contribute to an understanding of the phenomenon, and consequently none of the reports was excluded due to its quality [31,32].

## Findings

### Description of the Studies Included

The studies selected had been conducted in the USA [24,28,33–37], Canada [2,30,38,39], Sweden [40,41], the UK [16,42,43], China [44–46] and Austria [17]. Fifteen of the 21 studies dealt exclusively with patients [2,17,24,28,29,35,37–42,44–46], three exclusively with health professionals [33,34,36], and three considered the views of patients, health professionals and family members (see Table 2) [16,30,43].

**Table 2:** Description of studies included in the review.

Author(s)	Participants	Geographical location	Setting
Quill [28]	Patient with acute myelomonocytic leukaemia requesting physician-assisted suicide	New York, USA	Not specified
Bolmsjö [40]	Ten terminally-ill patients diagnosed with advanced cancer	Lund, Sweden	Lund University Hospital and a Lydiagarden centre for rehabilitation of cancer patients
Kade [29]	Patient with non-Hodgkin lymphoma requesting physician-assisted suicide	Not specified	Not specified
Mesler and Miller [33]	Thirty-five nurses, 9 social workers, 5 bereavement and/or volunteer coordinators, 3 nurse assistants, three executive directors, two chaplains, 1 regional manager, 1 medical director, and 1 physical therapist	USA	12 hospices
Lavery et al. [38]	Thirty-one men and 1 woman with HIV-1 or AIDS	Ontario, Canada	HIV Ontario Observational Database (HOOD), a provincial epidemiological database
Chochinov [30]	Patient with lung cancer with metastases to the liver, brain and adrenal glands.	Manitoba, Canada	Not specified
Chochinov et al. [2]	Twenty-three men and 27 women with terminal cancer	Manitoba, Canada	Urban extended care hospital housing a specialized unit for palliative care
Enes [16]	Eight terminally-ill patients (4 women and 4 men), 7 HPs (3 nurses, 1 doctor, 1 social worker, 1 chaplain and 1 physiotherapist) and 6 relatives (4 women and 2 men)	Surrey, UK	Hospice inpatient unit
Ganzini et al. [34]	Thirty-five physicians (8 women and 27 men)	Oregon, USA	Not specified
Coyle and Sculco [35]	Seven terminally-ill patients with cancer who had expressed a desire for hastened death	New York, USA	Urban cancer research centre
Volker et al. [36]	Nine oncology advanced practice nurses (from 39 to 55 years old)	Texas, USA	Members of the Oncology Nursing Society
Volker et al. [37]	Seven people with advanced cancer diagnoses	Texas, USA	Recruited via oncology advanced practice nurses
Mak and Elwyin [44]	Six advanced cancer patients who desired euthanasia while receiving palliative care.	Hong Kong, China	Unit followed the UK model ofmulti-disciplinary team palliative care with a multi-disciplinary team
Pearlman et al. [24]	Thirty-five patients who pursue a hastened death	Washington, USA	Patient advocacy organizations that counsel persons interested in PAS,hospices and grief counsellors
Chapple et al. [42]	Eighteen patients with terminal illness who discuss euthanasia and physician-assisted suicide	Oxford, UK	Interviews were contributions to the website DIPEX (Personal Experiences of Health and Illness; www.dipex.org)
Franklin et al. [41]	Twelve people aged over 85 years (10 women and 2 men)	Orebro, Sweden	Not specified
Pleschberger [17]	Twenty residents of nursing homes	Vienna, Austria	Not specified
Nissim et al. [39]	Twenty-seven ambulatory patients aged 45–82 years with advanced lung or gastrointestinal cancer	Toronto, Canada	Outpatient clinics at a large cancer centre
Brown et al. [43]	Fourteen clinical nurses, 3 general practice nurses, 8 patients with a diagnosis of a life-threatening illness and 5 carer	Scotland, UK	Not specified
Ho et al. [45]	Sixteen older Chinese palliative care patients with terminal cancer	Hong Kong, China	Terminal cancer patients receiving palliative care services in a major public hospital in Hong Kong
Ho et al. [46]	Eight men and 10 women (aged 44 to 92 years) diagnosed with stage IV cancer, with a life expectancy of no more than six months, living in the community either at home or in a long-term-care institution	Hong Kong, China	Patients enrolled in the out-patient palliative care programme of a major public hospital

### Overview of Themes

Three broad themes emerged from the synthesis of studies. The first theme was dignity mediated by the loss of functionality linked to the loss of control and of the value ascribed to one’s life. The second theme was dignity as identity, specifically in relation to self-identity and the impact of social factors. Finally, autonomy as the basis of dignity was understood as the desire for control over the dying process and the desire for self-determination. Table 3 indicates the presence of these themes in each of the studies, while Table 4 presents some quotations that represent each theme.

**Table 3:** Themes, subthemes and categories identified in each study.

Themes	Subthemes	Categories	Quill [28]	Bolmsjö [40]	Kade [29]	Mesler and Miller [33]	Lavery et al. [38]	Chochinov [30]	Chochinov et al. [2]	Enes [16]	Ganzini et al. [34]	Coyle and Sculco [35]	Volker et al. [36]	Volker et al. [37]	Mak and Elwyin [44]	Pearlman et al. [24]	Chapple et al. [42]	Franklin et al. [41]	Pleschberger [17]	Nissim et al. [39]	Brown et al. [43]	Ho et al. [45]	Ho et al. [46]	TOTAL
	Loss of control	Loss of bodily functions	✓	✓	✓	✓	✓	✓	✓	✓	✓	✓	✓			✓	✓	✓	✓		✓	✓	✓	18
**Dignity mediated by the loss of functionality**		Daily activities and circumstances	✓		✓		✓	✓	✓	✓	✓		✓		✓	✓		✓			✓	✓		13
	Value of one’s own life	‘Life without dignity no longer being worthy of living’	✓		✓	✓	✓	✓			✓					✓	✓		✓					9
		Quality of life		✓	✓		✓	✓	✓		✓	✓			✓		✓					✓	✓	11
		Body image	✓	✓	✓		✓			✓	✓	✓	✓	✓		✓	✓	✓						12
	Self-Identity	Loss of the self	✓				✓	✓		✓	✓	✓	✓	✓		✓								9
**Dignity as identity**		Loss of self-worth			✓		✓	✓		✓				✓		✓								6
		Dependent: being a burden	✓	✓	✓	✓	✓	✓		✓	✓	✓	✓	✓	✓	✓	✓	✓	✓	✓	✓		✓	19
	Social factors	Role						✓		✓	✓				✓	✓					✓	✓	✓	8
		Social identity: fear of being vulnerable		✓			✓	✓		✓	✓	✓	✓	✓	✓	✓	✓	✓		✓		✓		14
		Control over the body	✓		✓						✓						✓							4
		Control of pain	✓		✓						✓	✓	✓	✓	✓		✓			✓				9
	Desire for control over the dying process	Fear of suffering	✓		✓						✓	✓		✓	✓	✓	✓			✓				9
**Autonomy as a determining factor of perceived dignity**		Loss of functions	✓	✓	✓						✓			✓			✓			✓				7
		Control over the manner of death	✓		✓	✓	✓				✓	✓	✓			✓	✓			✓				10
		Independence: ‘do what I want’	✓		✓					✓	✓					✓	✓						✓	7
	Desire for self-determination	Right to choose	✓		✓	✓					✓					✓	✓			✓				7
		Decision making	✓		✓	✓					✓						✓						✓	6

**Table 4:** Some quotations from participants in the primary studies that illustrate each theme.

**Theme: Dignity mediated by the loss of functionality**	**Other related themes**
**Subtheme: Loss of control**	
“Um, the ability to perform simple things like, you know, going to the bathroom on your own and not through a bag, um, breathing with your own lungs, not dependent upon a machine to keep the body parts functioning, um being able to do anything, I mean as long as you can think then you can live, but if you can’t [sic] no longer even formulate a thought due to dementia or you know the ravages of the disease. You know, if you were to stand there in your former self, would you want to see yourself in that position? I know I wouldn’t. You get to the point where there’s no return, you know, I can understand somebody saying, well geez, you know, like I used to be somebody, but now, like I mean, you know, I’m no better than like a doll, somebody has to dress me and feed me and I guess it’s uh, I don’t know how to explain it, really” [38].	Dependency; Inability to perform daily living activities; Loss of identity
“Well it’s the same thing as living in your own home you know. You are your own person. And… and if anybody started telling me to do this do that you know, and you’ve got to be in bed at a certain time and you’ve got to have help being undressed and all that, I think… God Lord, that. . . would be the worst thing that could happen. That would really be losing dignity. I wouldn’t have any then” [2].	Loss of independence
“To the most—the simplest things, and when they were gone, he didn’t have a reason. . . So it wasn’t just the diarrhoea or the lack of driving; it was just losing, like, his definition—what his sense of vitality was. And when that was gone, then he was ready” [24].	Loss of identity; Loss of control
**Subtheme: Value of one’s own life**	
“I recognized only later that my patient’s goal was to be released from a life that had robbed her of her independence and dignity” [29].	Loss of control over one’s circumstances
“You talk about dignity. . . I’ve decided what I aim to do [I always wear make-up anyway, which I can’t do now], I’m going to make sure that I always have my make-up on; make sure everything is very clean, very tidy and my nails properly done. . .” [16].	Inner dignity; Physical image
“He told me that if all you can look forward to is your next enema, and you don't even like that much, what is the point of living?” [34].	Loss of the value of life
**Theme: Dignity as identity**	
**Subtheme: Self-Identity**	
“You’ve become a bag of potatoes to be moved from spot to spot, to be rushed back and forth from the hospital, to be carried to your doctors’ appointments or wheeled in a wheelchair, and it really does take away any self-worth, any dignity, or any will to continue to live” [38].	Loss of self-esteem; Feeling of being useless; Loss of the value of life
“I think to be calm is dignity. I’ve never been a calm person and I hate myself sometimes for that… I’m becoming more calm. I’ve control. . . To be in control of your emotions; that’s dignity” [16].	Inner dignity
“When I lost my hearing people started to ignore me. They didn’t treat me as a human being anymore and then when I lost my eyesight there was nothing left. I couldn’t go anywhere and couldn’t do anything. For example, I can’t hold the telephone and it’s impossible for me to put it back if no one helps me. My friends want me to contact them as well but I can’t without asking the girls and they have so much to do and are in such a rush so I forget to ask when they are in here” [41].	Loss of social recognition; Inability to perform daily living activities
**Subtheme: Social factors**	
Interviewer: “Would you feel that your dignity was taken away if your children needed to help you?” Participant: “Well, yes if I knew. . . I wouldn’t want them to take on the burden of doing that. That I have to depend on people just to look after me, to wash me, to take me to the bathroom and to cleanse… clean me up. . . I know this happens but I wish it didn’t happen to me” [2].	Dependency; Fear of being a burden on others
“It’s going to the loo. . . in privacy. . . with locks on the doors. . . and not leaving a mess in the loo. . . for other people to clean up. Em, trying not to make nasty smells. . . I know this sounds silly ‘cos. . . Its dignity…” [16].	Independence; Importance of privacy
“I’m not comfortable, and I can’t do anything, so as far as I’m concerned in quality of life I’m not living; I’m existing as a dependent non-person. I’ve lost, in effect, my essence” [24].	Dependency; Loss of the self
“I don’t want to be a burden to my family and I want to have a say in the kind of care that I receive… But life here is harsh. I have no say in what to eat or when to eat, and my life revolves around the working routine of staff members. I have to wake up and eat breakfast at five o’clock every morning because this is when the morning shift starts working” [45].	Fear of being a burden on others; Loss of independence
**Theme: Autonomy as a determining factor of perceived dignity**	
**Subtheme: Desire for control over the dying process**	
Participant: “If I’m going to be rolling around in my own faeces because I have no control, then forget it.” Interviewer: “Ok. Why—why is that such an important thing?” Participant: “Oh, it’s the dignity and wholeness of my body, as well as spirit. And, it is, it’s cruel too for others to have to do this when there’s no end in sight, other than death. To just, to clean me up. I just don’t want that. . . Dignity is that I have control over my body, when, when, not, not a virus that is going to take my life. I’m the one who’s going to decide when my life will end, not a virus, and not with great pain. Not anything else other than in, in my control. It is my control, my choice to do” [38].	Loss of identity; Desire for self-determination
“The patient said, ‘I don’t want strangers in my house. I’m doing fine. My wife’s taking care of me. I just don’t want people there 24 hours a day telling me what to do. And so I have had people refuse hospice because their understanding is that hospice takes control of their personal lives. They are very afraid of people coming in and they don’t want anybody to take over the role of their caretaker’” [36].	Desire for independence; Importance of privacy
“I will do things my way and the hell with everything and everybody else. Nobody is going to talk me in or out of a darn thing… What will be will be; but what will be, will be done my way. I will always be in control” [24].	Decision making
**Subtheme: Desire for self-determination**	
“So she was a control person. You know, we are talking big time control. . . You know, I am in charge here. She sort of self-directed her medical care. . . It was a control issue, not a pain issue. . .’I want to be in control of my destiny. I don’t want to go out as, you know, incontinent, in pain, crying, you know tearful person. I want to go out with some dignity’” [34].	Loss of functionality; Loss of the value of life; Desire for self-determination
“She just felt this was not dignified at all for a woman who had been in control all of her life. And she knew the end was near anyway. And she said, ‘I want to do it on my terms. I want to choose the place and time. I want my friends to be there. And I don’t want to linger and dwindle and rot in front of myself’” [34].	Desire for control over the end-of-life process; Desire for self-determination
“When I saw her she was very, very weak and very dehydrated. And again, I told her, I said, ‘Gee, you’re within a couple days probably of losing consciousness just from dehydration, and we could make sure that you just slept and did not suffer and it would just be a short time.’ She had the 15-day wait and she had 4 days before the medicine could be prescribed. And I told her that I didn’t think she would be able to do that unless she could solve the nausea and dehydration that she would last for 4 days consciously and to take the medicine. And she sort of struggled into a sitting position, asked her husband to get her a glass of water, and said, ‘I’ll get the fluids down somehow.’ And sort of forced… See, this is the paradox, this is where you learn that lesson about the control issue—she actually reversed the natural process to prolong her suffering, in order to be in control, to push the button herself” [34]	Desire for control over the dying process; Desire for self-determination

#### a) Dignity mediated by the loss of functionality

A key theme in the studies included was the perception of dignity being diminished due to a loss of functionality. In most of the studies this was reflected in the idea that the illness reduced control over one’s body and over daily activities and circumstances. An emerging sub-theme here concerned the value that patients ascribed to their life, which was often expressed in terms of quality of life [2,29,30,34,35,38,40,42,44–46].

Loss of control emerged both in relation to bodily functions and to daily activities and circumstances. A loss of control over one’s body (incontinence, loss of mobility, of cognitive functions, etc.) was a central feature in 18 of the studies [2,3,16,17,24,28–30,33,35,36,38,40,42,45,46]. Some patients stated that diminished functionality undermined their sense of dignity. Participants in many of the studies [2,15,22,26–28,34,36,39,41–43,45] described their inability to perform daily living activities, which was often related to feelings of hopelessness and of being useless.

In 11 of the studies this loss of functionality was associated with a diminished quality of life, and it was interpreted as a loss of the value of life [2,17,24,28–30,33,34,42]. By contrast, in those cases where patients highlighted an inner dignity linked to personal or spiritual values, a positive sense of dignity was maintained in spite of the loss of autonomy or control [30,40,43]. Being aware of this inner dignity enabled patients to feel that life still had value and meaning despite their current circumstances. These personal values were expressed in terms of having a positive impact on family ties [46] or in the form of religious or spiritual beliefs [17,30].

#### b) Dignity as identity

The second main theme that emerged concerned dignity as identity. In some way, dignity was seen as part of the patient’s identity and as something that was undermined by the dependency and fragility produced by the illness. This theme emerged with two sub-themes: self-identity (how the person sees him/herself), and social factors related to how the person believed he or she was seen by others.

Self-identity was related to physical image, the loss of self-esteem, and the loss of the self. The latter involved the perception that one’s identity or personal essence had been lost. Participants in many interviews described how the inability to look after themselves led to this feeling of a loss of self. In one study [38], some of the participants described the loss of their sense of dignity as a kind of disintegration of the self. Enes [16] referred to this notion as *being human* and *maintaining the individual self*.

For many participants their physical deterioration led to changes in their body. This was seen as altering their identity and as breaking the link with the person they had been prior to the illness. Some participants identified the loss of autonomy with being seen as “*vegetables*” [38,40,44] or with being treated as objects of little value, like “a bag of potatoes to be moved from spot to spot, to be rushed back and forth from the hospital” [38].

Another feeling associated with the loss of the self, and in some cases with physical deterioration, involved a loss of self-esteem. This loss of self-esteem was in turn associated with a feeling of being useless, since patients felt they had lost their social role, as well as with an awareness of their own vulnerability and their inability to manage daily living activities [2,29,37,38,42].

In all the studies the participants made reference to how their illness influenced their immediate social context. This concerned both the impact of their needing adequate care and attention, as well as how this affected their relationships and their perception of themselves.

In the majority of studies the idea of being a burden was related to the loss of social role, coupled with a feeling of being useless and a loss of the value of life. This occurred when the patient, who had previously occupied a certain role within the network of social relations, lost this status and felt that he or she was no longer seen as important or ceased to perform a set of functions due to the illness. Those patients who had occupied a key role within the family, who had once held a notable professional position or who had always valued their independence found it particularly difficult to have to depend on others and to lose their position of influence. Note, however, that in the study which Ho et al. [45] carried out in China, where a strong sense of the family unit still exists, this loss of social role led to a strengthening rather than an undermining of relations. The imminent death of a relative consolidated relationships within the family and was seen as an opportunity to pass on knowledge to younger generations.

A key issue in patients’ awareness of their dependency was when they needed a professional or relative to help with personal hygiene, eating, getting dressed or using the toilet [2,40,41,44]. In such cases this help was seen as humiliating and as an intrusion into one’s privacy. Some patients expressed their fear of becoming a financial burden on the family, and, especially, of requiring kinds of help that they would never have wished to need [2,16,30,38,42].

Relatives and patients stated that health professionals could influence the patient’s perceived dignity through the care, empathy and attitude they showed [2,16,30]. The wife of a man receiving ‘dignity therapy’ [30] said that one objective for professionals should be “helping the patient to feel that he is still of value”, and she said that her and her husband’s dignity had been maintained as a result of the care they had received in the hospital. By contrast, in studies such as that by Pleschberger [17] or Pearlman et al. [24] some patients said that they had been treated like objects, highlighting how this had made them feel ashamed. In 18 studies, mention was made of the fear of being vulnerable and fragile in relation to one’s surroundings, and this was related to seeing oneself as dependent or as an object of contempt [2,24,34,35,38,42]. Some patients referred to how upsetting it was to think that they would be remembered as being useless and incapable.

#### c) Autonomy as a determining factor of perceived dignity

The third theme encapsulated two situations: desire for control *over the dying process* and desire for *autonomy in the form of self-determination*. Initially, the loss of control over one’s life and circumstances was linked to the loss of control over the body and basic functions [28,29,34,42], to the fear of suffering [24,28,29,34,37,39,42,44], and to control over how one would die. Subsequently, however, it was related to more inner aspects, that is, to the patient’s self-perception and to the feeling that life like this was not worth living.

A view shared by most participants was that their dignity had been diminished by the loss of control over their life and circumstances: physical functioning, pain, suffering and how they would die [2,3,16,17,24,28–30,33,35,36,38,40,42,45,46]. For many of these patients, the fact that they were no longer in charge of their own body was experienced as undermining their dignity and as stripping life of its value. Although a loss of physical functioning, for example, is a common occurrence among patients with advanced disease, the participants in the studies included emphasized a strong desire for control and autonomy. These patients expressed feelings of impotence and frustration associated with the awareness of their progressive and inevitable deterioration, with a suffering that seemed meaningless.

In some studies [28,42] the medical impossibility of controlling pain, the adverse effects of treatments such as chemotherapy [26], or the cognitive effects (delusions, personality changes, etc.) of certain drugs were equated with an ‘undignified death’.

The fear of suffering emerged in response to uncertainty about the future and irreversible deterioration. This fear was associated with feelings of anguish and despair, as well as with the corresponding fear of making others suffer. In the majority of cases this fear of suffering manifested as the expectation that the future would bring unbearable physical, emotional or existential pain. In some studies this kind of response to expected suffering was related to a previous negative experience involving the death of a relative or friend. Upon recalling this pain and suffering some patients reacted with fear or resistance as they did not wish to go through the same process [24,35,44].

In a similar vein, in those studies that explored the desire or wish to hasten death or the motivations that led patients to request physician-assisted suicide or euthanasia, this wish was related to the perception of a future that would be worse than death itself [44]. In other words, death here is seen as a way of putting an end to suffering. For example, in the case studies of Quill [28] and Kade [29] both patients had requested assisted suicide and this decision was taken prior to their finally losing control over their body or experiencing unbearable pain. What stood out here was their fear of suffering and of the pain to come, and their belief that the dying process would be intolerable.

The desire for self-determination can be regarded as a basic principle among those patients for whom the right to choose and to make their own decisions was of paramount importance. Two categories emerged here: the desire for independence, in the sense of ‘Do what I want’, and the right to decide.

Patients who defended the notion of self-determination were described as people showing a strong and independent character in the different areas of their lives [24,34]. Hence their desire to have control over how they die is regarded both by themselves and by the authors of the studies concerned as being a natural consequence of their character and of their approach to life. In other words, they do not wish their lives to be subject to external rules that restrict their freedom, or to be determined by aspects of their illness or the need for help from others [16,24,28,29,34,42,46].

Common to these patients was a belief in the right to choose how they would die and what treatments they would accept or refuse. Likewise, people who showed a strong desire for control appeared as active participants in the process of their medical care, and they were not prepared to accept paternalistic attitudes on the part of health staff. The possibility of a hastened death was seen as the ultimate opportunity for control and freedom that a patient could have [34,35,42]. Thus, being able to exercise this freedom was interpreted as a way of rising above one’s circumstances, including—paradoxically—their imminent death.

## Discussion

This systematic review and interpretative synthesis confirms, from a theoretical and empirical point of view, that patients’ perceived dignity at the EOL is related to their sense of autonomy and ability to control physical functions and their immediate surroundings. The illness experience, the transformation of identity and the influence of the social context are aspects that have been referred to in numerous settings [46–53], and in this regard the present synthesis, which takes multiple factors into account, can help to clarify the different ways in which the concept of dignity has been used in relation to autonomy.

The first theme to emerge from our analysis, namely perceived dignity mediated by loss of functionality, is a constant feature in research on the illness experience. In the EOL context, and regardless of whether the specific focus of a study was perceived dignity and control, the wish to hasten death (WTHD) or attitudes towards physician-assisted suicide (PAS) and euthanasia, this theme was an inevitable starting point given that the experience of dignity is determined by the patient’s illness. In studies such as those by Chochinov et al. [2], Volker et al. [37], Pearlman et al. [24], Brown et al. [43] and Ho et al. [45] the loss of functionality was expressed in terms of illness-related problems or experiences that have an impact on perceived dignity. In other studies the loss of functionality was expressed directly as ‘disintegration’ [38], ‘loss of the self or essence’ [24,38] and ‘perception of suffering for self’ [44].

The second theme identified, namely dignity as identity, was defined in terms of self-identity and in relation to how patients perceived they were seen by others. Numerous authors have highlighted the impact that the physical transformation produced by illness can have on personhood [5,16,48–52]. Street and Kissane [5] stated that ‘dignity is embodied’ since individuals know themselves and are known through their corporality. Hence the illness experience can imply a separation between an individual’s sense of him or herself as a person and the ‘altered’ body that is no longer recognized as one’s own [50]. This phenomenon was referred to by Franklin et al. [41] as ‘the unrecognizable body’.

The question of identity is also linked to certain emotions such as self-esteem that involve an appraisal of oneself [2,16,24,29,37,38]. Another common emotion is the fear of ‘losing oneself’, that is, of ceasing to be the person that one once was and, therefore, of letting others down. Shame may also be felt as a result of the loss of privacy inherent to the need for help with daily activities, or due to the lack of control over oneself and one’s everyday life.

It should also be noted that the view of self cannot be separated from how the person feels that he or she is seen or treated by others. Chochinov et al. [2], as well as those studies that have applied his model of dignity, place particular emphasis on what is referred to as the ‘social dignity inventory’, which has to do with how the quality of interactions with others influences a person’s sense of dignity. In studies of the WTHD, euthanasia or PAS, patients often refer to the fear of making others suffer and regard the wish to end their own life as an altruistic gesture [35,53]. The category of ‘being a burden on others’ has also been highlighted by several studies, and at times is reinforced by patients’ fear of being seen as vulnerable or incapable by their loved ones. These ideas would likewise be related to the aforementioned ‘loss of self’. Interestingly, however, the EOL situation has also been understood as an opportunity to strengthen the sense of ‘connectedness and belonging’ with respect to close friends and relatives [16,24,44].

The final theme that emerged from our systematic review was autonomy as a determining factor of perceived dignity. In the literature on the WTHD, euthanasia and PAS, one repeatedly finds support for the idea of a ‘dignified death’, the premise being that a person’s dignity depends on his or her ability to maintain autonomy and control. A meta-ethnography of the WTHD [54] found that this wish arose “as a kind of control over one’s life”. In the present analysis, the sub-theme ‘desire for control over the dying process’ would be linked to this wish to maintain a degree of control over certain aspects of one’s life, which does not necessarily imply taking some kind of action to hasten death. However, the second sub-theme, ‘desire for self-determination’, would be directly related to an explicit wish and willingness to end one’s life.

### Explanatory Model

**Fig 2:**
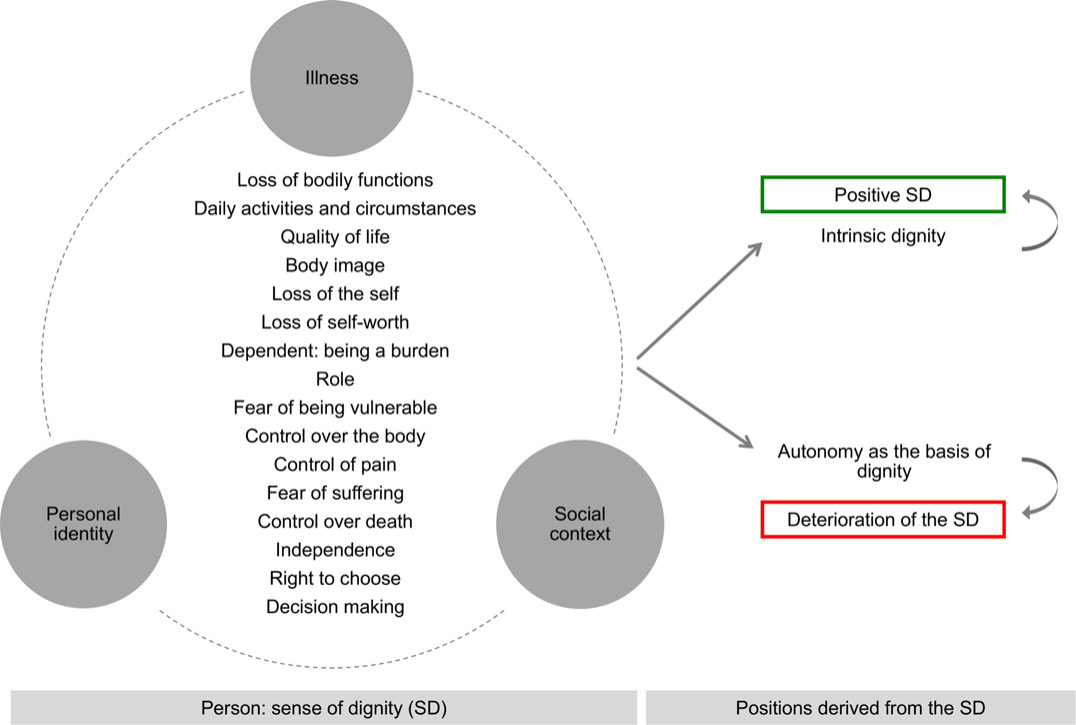
Explanatory model. This figure shows the dynamic integration and synthesis of the themes, subthemes and categories emerging from the lived experience of perceived dignity, autonomy and control.

This synthesis provides an explanatory model that integrates in a dynamic way the various themes explored in the study (see Fig 2). Based on this model, one could say that the experience of all the participants was shaped by their illness experience, the social context and the impact of illness on their personal identity.

At the most descriptive level of analysis (process of identifying categories) we found that the majority of patients related the perceived loss of dignity to various factors: ‘loss of bodily functions’ in the context of ‘daily activities and circumstances’; a diminished sense of the ‘value of one’s own life’; loss of ‘quality of life’; a transformation of ‘body image’; a ‘loss of the self and self-worth’; feeling ‘dependent’, ‘vulnerable’ or that one was ‘a burden’ in the social sphere. The perception of dignity was also influenced by a ‘fear of suffering’, especially in relation to the ‘desire for control over pain’, ‘over the body’ and ‘over the manner of death’, as well as a strong desire for independence, for the ‘right to choose’ and for a ‘decision-making’ capacity.

At the more interpretative level of analysis, however, there was a consensus among the research team that these categories could not be defined as isolated elements. Rather, and given that the person is a holistic being, these categories need to be considered from a more integrative perspective in which the physical experience of illness is inseparable from its influence both on personal identity and on others (i.e. on the social context). For example, the analysis suggests that perceived dependency is the primary mediator of a diminished sense of dignity. In this case, dependency cannot be understood in isolation from the impact of the illness (which is the root cause of this dependency), from the new care relationship that emerges in the patient’s immediate surroundings, or from the construction of a new identity that is determined by the illness and a new way of life marked by anxiety, frustration, fear and uncertainty, etc.

Within this framework, two contrary positions can be observed. Those patients who emphasized the awareness of an internal or intrinsic sense of dignity maintained a positive view of themselves in the face of their illness [16,36,40,45] By contrast, patients whose sense of dignity was based on values such as autonomy, the ability to control their circumstances or quality of life found that their dignity was undermined [24,28,29,34,38,42].

One of the key themes to emerge from the present analysis concerned the desire for control over the dying process among patients whose sense of dignity was based on autonomy. Although there is some controversy regarding what motivates people to request euthanasia or assisted suicide, the results of this synthesis indicate that, when faced with a lack of control over pain, the inability to enjoy everyday life or the possibility of being a burden on others, then people with a strongly independent and ‘controlling’ nature are more likely to express a wish to hasten their death.

By using the methodology developed by Noblit and Hare [26] we were able to include a heterogeneous set of studies involving different methodologies, such that the final sample comprised 400 participants. In relation to the importance ascribed by Noblit and Hare to recognizing not just similarities but also points of difference, there was one concept that only featured in the study by Ho et al. [45], namely ‘transgenerational unity’. This category reflected the importance given in Chinese culture to the connection between different generations, this being regarded as part of spiritual unity within the family. The study by Brown et al. [43] was likewise the only study in which health professionals stated that when a patient or his/her relatives perceived a sense of burden, then this was the time to implement strategies aimed at increasing the patient’s independence and at providing the care needed to ensure that he or she felt well treated.

### Limitations

One limitation of the present research is that although the majority of studies reviewed included people with advanced cancer, the final sample comprised participants with different symptoms and disease processes, and this may hamper the transferability of results. Furthermore, none of the studies included had the specific aim of describing the relationship between dignity and autonomy, and given the findings of Sandelowski et al. [55] this could reduce the frequency effect size. Another potential limitation is that although we designed a sensitive and specific search strategy, the subtlety with which the concepts defined by our search terms appear may have limited our findings in the databases used. Finally, mention should be made of the small number of countries in which the reviewed studies were conducted: the 21 studies included covered only six countries. With the exception of China, there may therefore be a degree of cultural homogeneity.

### Implications for Practice

Given the intrinsic value of dignity, the fact that a person’s sense of dignity can be influenced by a range of external factors suggests that specific steps need to be taken to preserve it. The different therapies or models of dignity that have been developed to date illustrate the positive impact that these interventions can have on the individual concerned [2,43]. Clearly, these preventive or therapeutic measures must take into account the different aspects or areas of life on which a patient’s sense of dignity is based. In this regard, the results of this systematic review suggest that the loss of functionality coupled with dependency and the feeling of being a burden on others is one of the factors most likely to lead to a diminished sense of dignity. Consequently, anticipating possible frustration and empowering patients in those areas where they can make their own decisions and be more autonomous may prove to be beneficial.

The results of the review also suggest that health professionals need to be aware of the importance of taking into account the intrinsic or internal dignity of the people for whom they care. In fact, such awareness is important for caregivers, relatives, and even society as a whole, since on different levels they each can influence the extent to which an individual feels dignified or not. This way of seeing the other may favour the wellbeing and self-perception not only of the patient but also of the family members and professionals who are involved in his or her care. Consequently, there is a need to develop individualized treatment plans that foster this way of treating and caring for patients, and also to ensure that professionals receive adequate training in how to implement them.

### Lines of Future Research

Given the influence of the family and social context, the various models of dignity that focus on the patient [2,3,43,56] could usefully be complemented by research into the strategies that family members could best implement in order to safeguard the perceived dignity of their loved ones. Likewise, although some quantitative research has examined patients’ desire for control at the EOL, there are, to our knowledge, no studies exploring the experience and meaning attributed to this desire by patients themselves. A deeper understanding of this wish for control could help in the design of interventions to ameliorate this loss of self-identity or dignity in relation to autonomy and the WTHD.

## Conclusion

Although dignity can be considered to be an intrinsic feature of human life, the results of this systematic review highlight how it is a complex, multifaceted and dynamic concept, one that is closely linked to the notion of personal identity. A more in-depth understanding of the experiential context of patients at the EOL may help to ensure that they are not reduced to their circumstances. Furthermore, given that the sense of dignity is readily influenced by a number of external factors, it is important to develop care plans that address the areas of life on which a patient’s dignity is based, and thus contribute to an improved quality of life.

## Supporting Information

S1 Table Methodological Quality of included studies assessed with CASP: qualitative research checklist. (DOCX)

S2 Table Quotations from participants in the primary studies that illustrate each theme. (DOC)

S3 Table Methodological Quality of the systematic review assessed with AMSTAR (a measurement tool to assess systematic reviews). (DOC)
