# Supplementary material for: Patient Perspectives of Dignity, Autonomy and Control at the End of Life: Systematic Review and Meta-Ethnography
Source: PLoS One. 2016 Mar 24;11(3):e0151435. doi: 10.1371/journal.pone.0151435 (PMC4806874; doi:10.1371/journal.pone.0151435)
Supplement: S2 Table — (DOC) [file pone.0151435.s002.doc]

**S2 Table**. Quotations from participants in the primary studies that illustrate each theme

| **Theme: Dignity mediated by the loss of functionality** | **Other related themes** |
| --- | --- |
| **Subtheme: Loss of control** |
| Interviewer: “Ok and what do you mean by dignity?” Participant: “Um, the ability to perform simple things like, you know, going to the bathroom on your own and not through a bag, um, breathing with your own lungs, not dependent upon a machine to keep the body parts functioning, um being able to do anything, I mean as long as you can think then you can live, but if you can’t [sic] no longer even formulate a thought due to dementia or you know the ravages of the disease. You know, if you were to stand there in your former self, would you want to see yourself in that position? I know I wouldn’t. You get to the point where there’s no return, you know, I can understand somebody saying, well geez, you know, like I used to be somebody, but now, like I mean, you know, I’m no better than like a doll, somebody has to dress me and feed me and I guess it’s uh, I don’t know how to explain it, really.” [38] | Dependency Inability to perform daily living activities Loss of identity |
| “I am so angry when I have only one side that works, when I have been a hands-on person all my life. I get angry and frustrated because I can’t do things that used to be so easy for me to do.” [30] | Inability to perform daily living activities |
| “But I guess that [dignity] is something that I’ve lost... [Knowing] it’s going to get worse. I know it will [be] quick, or I hope so. When I get to the point where I can’t go to the bathroom anymore or where I can’t... all the things that I still have control over... I suppose maybe you get to the point where you have to be all plugged into a whole bunch of stuff and tubes and everything else. But maybe you go so far down the road those tubes... Maybe it doesn’t matter anymore. But I’m not looking forward to that day... You know, I just heard in the last couple of minutes you sort of... [I’m] worried and scared. But it’s just something that you thought about the timing and circumstance of... I guess I worry about it more than I think...Yeah. I don’t know what’s in store and I think my fate is, you know, the time and what it brings me sort of thing. It doesn’t make it easy.” [2] | Inability to perform daily living activities  Fear of future suffering |
| “Well it’s the same thing as living in your own home you know. You are your own person. And… and if anybody started telling me to do this do that you know, and you’ve got to be in bed at a certain time and you’ve got to have help being undressed and all that, I think… God Lord, that... would be the worst thing that could happen. That would really be losing dignity. I wouldn’t have any then.” [2] | Loss of independence |
| “She was totally bedridden. She was messing her sheets and stuff like this, and Mother just—I mean, she’s just—she was a very fastidious person. And she just—she—well, basically, she thought the quality of her life was appalling. She couldn’t do anything. All she could do was lie in bed.” [24] | Loss of quality of life |
| “To the most—the simplest things, and when they were gone, he didn’t have a reason... So it wasn’t just the diarrhoea or the lack of driving; it was just losing, like, his definition—what his sense of vitality was. And when that was gone, then he was ready.” [24] | Loss of identity Loss of control |
| “The nurse was over there, basically, manually helping her along… And she just said, ‘This is not worth it’… And a lot [had] to do with her as a person where she just was so independent. The whole idea of nursing to her was just abhorrent.” [24] | Loss of independence Social identity: perceived vulnerability |
| “I am afraid of the day when I can’t even get out of bed.” [41] |  |
| "No one told me that I had cancer in the beginning, not my doctor or my children", said an 82-year-old home-dwelling female patient. ‘They didn’t want me to be upset, I guess, but I have a right to know. I finally got the answer from a second doctor that I consulted, and honestly, I wasn’t upset to hear the news. I mean I have lived a long life and all my children have grown up. I am not afraid of dying... but I don’t want any more painful medical procedures. After talking to my attending nurse, I signed off my advanced care directives so that my family members don’t have to make those difficult decisions for me.’” [46] | Loss of control over information Decision making |
| **Subtheme: Value of one’s own life** | |
| “I recognized only later that my patient’s goal was to be released from a life that had robbed her of her independence and dignity.” [29] | Loss of control over one’s circumstances |
| “My patient was suffering at the core of her being without agonizing pain, anorexia, or night sweats. She had become increasingly dependent on others for virtually all activities. Her dignity, her self-esteem had been stripped away. The vitality of her being had passed. Yes, her life, as she defined it, had become futile.” [29] | Dependency Loss of self-esteem Inability to perform daily living activities Loss of the value of life |
| “You talk about dignity... I’ve decided what I aim to do [I always wear make-up anyway, which I can’t do now], I’m going to make sure that I always have my make-up on; make sure everything is very clean, very tidy and my nails properly done...” [16] | Inner dignity Physical image |
| “He told me that if all you can look forward to is your next enema, and you don't even like that much, what is the point of living?” [34] | Loss of the value of life |
| “It would be a comfort to me to think that when I’ve come to a point where I’m clearly dying, you know there is no further treatment available for me and if I am in lots of discomfort, I would like to be able to say, ‘Can you get my kids to come and see me,’ and maybe, I don’t know, my friend, that’s a minister or whatever and say goodbye. And then [er] you know can you just do what has to be done, give me an overdose of morphine or whatever it is. Because actually my Mum was in hospital for about three months before she died. And she had sort of raging osteoporosis having taken lots of steroids for another condition. And she was in terrible pain and she had made a living will actually. [Um] And they had to give her so much morphine in the last few weeks, to be honest she was talking rubbish and coming up with ideas. She told us there was something she wanted to tell me and that she had murdered twelve children. And I said, ‘Oh Mum, you know that is your mind playing tricks because of the drugs. You haven’t murdered anybody’. But she still insisted that she had. And I just wish that she could have gone a month or two earlier before she went through all that indignity that is dying really. [um] And I would feel the same about myself [...]. I just think that when you’ve come to that stage, only you know when that is, how bad that has to be, you don’t need to go through the physical indignities of throwing up, being smelly, being incontinent, whatever it might be.” [42] | Loss of functionality Fear of future suffering Desire to hasten death |
| “I can’t do anything. Since they amputated my legs I just wheel around in my wheelchair.” [41] | Inability to perform daily living activities |
| “I just lie here; you see I can’t move, my body just won’t let me. I used to be very active and travel a lot.” [41] | Loss of functionality |
| “If you lose your mind you will soon lose in value.” [17] | Loss of the value of life Loss of functionality |
| “Dignity is really about living a normal life... being able to eat what I like, do things that I enjoy, and be with my family give me dignity.” [45] | Performing daily living activities Independence |
| **Theme: Dignity as identity** | |
| **Subtheme: Self-Identity** | |
| “You’ve become a bag of potatoes to be moved from spot to spot, to be rushed back and forth from the hospital, to be carried to your doctors’ appointments or wheeled in a wheelchair, and it really does take away any self-worth, any dignity, or any will to continue to live.” [38] | Loss of self-esteem Feeling of being useless Loss of the value of life |
| “I think to be calm is dignity. I’ve never been a calm person and I hate myself sometimes for that… I’m becoming more calm. I’ve control... To be in control of your emotions; that’s dignity.” [16] | Inner dignity |
| “She [patient] used to be a pretty dynamic lady. She has a brain tumour and it - she had everything because of her high steroids... She put on so much weight that her body image... changed... and she finds that the way she eats is so grotesque… way of eating. She knows that she’s wrong and she’s like: ‘I lost - I lost my body image and I don’t care.’” [16] | Physical image |
| “Said [to the patient], ‘Well, what do you want to do?’ And she said, ‘Well, what am I allowed to do?’ And I said, ‘What you want to do’. She said, ‘You mean I can decide what I want to do?’ I said, ‘Yeah, what do you want to do?’ She said, ‘I want to take all these tubes out and I want to go home and to go back to church and do clog dancing.’ I said, ‘Well, what if… not your spirit, not your soul… but your physical body isn’t going to do that? What else is important?’” [36] | Inner dignity |
| “Everybody needs their space. Even though you need to be compassionate, still respect their space. When I’m really in pain, just totally a mess, let me have my space. I don’t know about other people, but that’s one thing about me. When I’m really, really bad, I just want to be left alone.” [37] | Need for independence  Importance of privacy |
| "I've seen patients yelling in pain and they were suffering… so intolerable… Just to hear them was very suffering… It must have been so unbearable to have to yell like that. If they could endure it, they wouldn't have yelled." [...] Someone with a tube ticking up the bottom, a tube sticking into the nostrils, another somewhere else- I mean, what for? Can you save them?" [44] | Loss of the value of life Suffering |
| “When I lost my hearing people started to ignore me. They didn’t treat me as a human being anymore and then when I lost my eyesight there was nothing left. I couldn’t go anywhere and couldn’t do anything. For example, I can’t hold the telephone and it’s impossible for me to put it back if no one helps me. My friends want me to contact them as well but I can’t without asking the girls and they have so much to do and are in such a rush so I forget to ask when they are in here.” [41] | Loss of social recognition Inability to perform daily living activities |
| “I am highly pleased anyway, as long as you can help yourself it is wonderful in this place. But when you cannot help yourself and get ill then it is not that jolly any longer.” [17] | Loss of the value of life |
| “I stopped worrying about this and that as I really have little control over things... all I am concern about is living in the moment and spending time with my family.” [45] | Inner dignity Living the present |
| **Subtheme: Social factors** | |
| “I wish I didn't have to vomit, pass waste, and so on uncontrollably. Looking disgusting is never dignified. Being sad, angry, scared, and so on hasn't got anything to do with dignity if you the staff, I think you can keep your dignity even if you do look distinguishing. I'm just now wondering whether perhaps losing one's dignity is like getting into situations where you would normally feel embarrassed and ashamed. There probably aren't too many situations where you can lose your dignity in the eyes of those closest to you. Getting to know one another is important.” [40] | Loss of functionality Inner dignity Social identity |
| “I'm scared of ending up like a vegetable. I can't even think about that - being so dependent that I can't manage anything… helplessness and pain. There's some hope in being able to go through a difficult crisis and pull back again. Think what it must be like to need help going to the toilet, help washing yourself, perhaps not being able to talk, express oneself. Suicidal ideation: I'm afraid of ending up just lying there, not being able to do anything, and just waiting to die. In that case, I'd rather take an overdose.” [40] | Loss of functionality Fear of dependency Desire to hasten death |
| “Well I think you lose your dignity when you’ve got to be fed. I mean you feel so foolish. You know I really did not want to be fed. But they kind of... they forced me. Having to be waited on and looked after and maybe even fed. I don’t know. But I… oh I would hate that. I like to be independent.” [2] | Dependency Loss of identity |
| “Actually for us it's been fairly few patients that ring so clearly in my mind, where they were just in pain until the end. But even the people that aren't in pain have to go through so many indignities - you have people bathing you, and touching you, and there's no privacy. I wouldn't want to go through it; no way.” [33] | Loss of functionality Dependency |
| “And I was on the commode and I had to be wiped and I just about cried my eyes out because of... you know, I never felt... I said to the nurses, God, who would have ever thought it would ever come down to this. I got these diapers or whatever it is that they call it... And that’s presenting a problem. I don’t like to think of myself as that. Things like that. That’s my dignity and it comes down to types of things like that really...So I get mad.” [2] | Loss of functionality Dependency Suffering in relation to one’s vulnerability |
| Interviewer: “Are there other things that would take dignity away from you?” Participant: “Having that young woman come in here the other day was very, very hard on me. Or to ask her to do anything for me, I find it very hard to ask her anything. I don’t know how to describe it. For one thing, I’ve never had too much self-esteem I suppose and I always preferred to meld into a wall. I felt more comfortable there… Uh, things like not being able to go to the washroom by myself. Oh to me, that would take everything away from me because I am so modest.” [2] | Loss of functionality Dependency |
| Interviewer: “What would take dignity away from your life?” Participant: “Oh my God, maybe putting me on the toilet seat. These are private things, you know. I still feel like I like my privacy. Even for my sleeping in, it’s kind of embarrassing if I’m still sleeping if they come downstairs. You know I’ve got to be... I’ve got to be up first and get dressed and be here...” [2] | Dependency  Importance of privacy |
| Interviewer: “Would you feel that your dignity was taken away if your children needed to help you?” Participant: “Well, yes if I knew... I wouldn’t want them to take on the burden of doing that. That I have to depend on people just to look after me, to wash me, to take me to the bathroom and to cleanse… clean me up... I know this happens but I wish it didn’t happen to me.” [2] | Dependency Fear of being a burden on others |
| “It’s going to the loo... in privacy... with locks on the doors... and not leaving a mess in the loo... for other people to clean up. Em, trying not to make nasty smells... I know this sounds silly ’cos... Its dignity…” [16] | Independence Importance of privacy |
| “Telling someone that... that maybe their vision is impaired and they may not be able to read again is a devastating blow for someone but it doesn’t have any knock on implications from the point of view, necessarily of their dignity. But telling someone that they won’t be able to em, toilet themselves, that seems really sort of - very devastating for someone...” [16] | Loss of functionality Dependency |
| “Professional W: `Anything that affects their, I suppose *capacity* to self-care and to be ever increasingly dependent on others would have an impact on the fact there’s a dignified human being.’” [16] | Loss of functionality Dependency |
| “After a while, your family, who you love so dearly, will remember you as a washed-out role model... It will remind them of what they have to go through, the lack of strength, the weakness, and so forth.” [35] | Suffering in relation to one’s vulnerability |
| “When I’m in the hospital, I don’t like to have tubes in me and I don’t like people coming in and seeing those things in me. I want them hidden because it’s very uncomfortable for everyone. The last time I was in the hospital, I had a visitor with me. And it wasn’t a family member. The nurse came in to check my [urinary] catheter to see how much it drained and to empty it. It just caught me so off guard. I didn’t appreciate that, because it’s like sharing my urine with other people.” [36] | Importance of privacy Social identity |
| “As far as dignity’s concerned, it’s just like last night [after falling and requiring paramedic intervention]. My dignity was hurt more than the bruises I’ve got. My pride and everything was gone at that point. It’s been hard having to stay with my son. That’s a great break in the independent thing. When I moved down here, my son said, ‘I never ever expected to have a wheelchair in my front room. I just can’t accept the fact that I cannot work anymore and have to live with him because I can’t afford to live on my own because I can’t afford the help.’” [36] | Dependency |
| “I said, ‘No, you can’t give Neulasta on the same day as Gemzar® [gemcitabine hydrochloride, Lilly Oncology, Indianapolis, IN]; I’m going to have to come back tomorrow.’ And so the next morning, I called another nurse, and she said, ‘No, absolutely not; we don’t give Neulasta with Gemzar.’ This can be quite scary. Because you take a healthy patient like me and kill ’em. It really makes me mad. I’m really considered what I think is a pretty difficult patient because I’m so on top of what has to be done to me.” [36] | Decision making |
| “I can’t move, just lie here... feeling like a vegetable... a useless person… needing people to feed me.”  “I just don’t want to endure these psychological effects… So much suffering... I have had the pain for four years… So many psychological effects… How can I bear it?” “Wish to live but can’t live; wish to die but can’t die.” [44] | Dependency Loss of the value of life Suffering in relation to one’s vulnerability Identity |
| “I’m not comfortable, and I can’t do anything, so as far as I’m concerned in quality of life I’m not living; I’m existing as a dependent non-person. I’ve lost, in effect, my essence.” [24] | Dependency Loss of the self |
| “He said that he doesn’t want to just turn into this vegetable kind of person where you’re not aware of what’s going on, and that everybody around you is affected; everybody’s having to take care of you, feed you, clean you, give your medication.” [24] | Dependency Loss of the self |
| “One patient reported, ...not wanting to be seen by those that love me as ‘this skin-and-bone frail, demented person’. In other words, ‘I don’t want that image of me for me, and I don’t want that image to be kind of a last image that my daughters and loved ones have of me’. And that’s just a dignity issue.” [24] | Suffering in relation to one’s vulnerability Social identity |
| “I need help with everything; I can’t even go to the bathroom by myself.” [41] | Inability to perform daily living activities |
| “It’s horrible not being able to take care of myself. I can always get help but it’s horrible to wake up when you wet your own bed. Everything you do you’re dependent on others. For example, when you need to go to the toilet. It doesn’t feel good to ask for help going to the toilet, just like babies.” [41] | Inability to perform daily living activities Suffering in relation to one’s vulnerability |
| “I’m scared of being dependent, tied to the bed and not being able to take care of myself, but there is no point in worrying. I try to enjoy the moment. The sun is shining or a flower is blooming… things like that.” [41] | Suffering in relation to one’s vulnerability Social identity |
| “[…] And that is a horror for me, that I might possibly have to lie in bed from morning to evening and from evening to morning and that I am dependent on another person to make every handhold that is important for my care and support. That I cannot any longer do everything alone, that is a terror for me. And I say, quite honestly… when I pray, I pray that I may also be spared that.” [41] | Dependency |
| “You know they just demand to take over all the time...they could ask.” [39] | Decision making |
| “I am totally dependent on my wife to carry me down the stairs, out of my home and into the outside world... My friends just seemed to have disappeared in my life.” [45] | Loss of social recognition  Dependency |
| “When I first got sick and was admitted to the hospital, I came out of surgery feeling very weak...I couldn’t eat under my own strength and the nurses came by a few times offering to feed me. I have never been fed since childhood, but if life has come down to this, I would want my daughter to do it.” [45] | Dependency |
| “When I got out of the hospital my daughter became very conscious and restrictive of my diet. She didn’t allow me to eat fatty food or go out for tea at my favourite restaurant... I got so angry at one point that I said, ‘‘I am going to die soon, so just let me eat what I want, and let me die a happy and fulfilled woman.” [45] | Loss of independence |
| “I don’t want to be a burden to my family and I want to have a say in the kind of care that I receive… But life here is harsh. I have no say in what to eat or when to eat, and my life revolves around the working routine of staff members. I have to wake up and eat breakfast at five o’clock every morning because this is when the morning shift starts working.” [45] | Fear of being a burden on others Loss of independence |
| **Theme: Autonomy as a determining factor of perceived dignity** | |
| **Subtheme: Desire for control over the dying process** | |
| “It was extraordinary important to Diane to maintain control of herself and her own dignity during the time remaining to her […] Knowing of her desire of independence and her decision to stay in control, I though this request made perfect sense.” [28] | Desire for control over the dying process Desire for independence |
| “She was convinced she would die during the period of treatment and would suffer unspeakably process” [from hospitalization, from lack of control over her body, from the side effect of the chemotherapy and from pain and anguish]. [28] | Fear of suffering Loss of control over functionality Loss of quality of life |
| “She was frightened to be leaving, but that she would be even more terrified to stay and suffer.” [28] | Fear of future suffering [more than of death itself] |
| “It was also evident that the security of having enough barbiturates available to commit suicide when and if the time came would leave her secure enough to live fully and concentrate on the present.” [28] | Security of being in control |
| Participant: “If I’m going to be rolling around in my own faeces because I have no control, then forget it.” Interviewer: “Ok. Why—why is that such an important thing?” Participant: “Oh, it’s the dignity and wholeness of my body, as well as spirit. And, it is, it’s cruel too for others to have to do this when there’s no end in sight, other than death. To just, to clean me up. I just don’t want that... Dignity is that I have control over my body, when, when, not, not a virus that is going to take my life. I’m the one who’s going to decide when my life will end, not a virus, and not with great pain. Not anything else other than in, in my control. It is my control, my choice to do.” [38] | Loss of identity Desire for self-determination |
| “Reasons for requesting assisted suicide more often had to do with future fears of suffering rather than current suffering: ‘He was very fearful of what the end might be like. He was worried about the pain. He was worried about the shortness of breath. And yes, he was very up-front about being afraid to die in misery.’” [34] | Fear of suffering |
| “His family said what he really wants is control, he doesn’t want to lose control.” [34] | Desire for control |
| “The option to extract oneself from an untenable situation through a hastened death sometimes was expressed as an “if-then” proposition; for example, ‘If the pain gets worse, then I want to be dead.’ ‘Pain is my biggest fear. It puts me in darkness and a lack of will to go forward and a desire to die… The pain wants me to have a vehicle to just, just stop my life.’” [35] | Fear of suffering |
| “There’s a fear about having pain and losing dignity when they die. Patients say, ‘When I die, I hear that I may wet myself, or mess myself.’ And that’s a big issue.’ Another APN observed that her patient’s increasing weakness ‘was the thing that took a lot of her control away’ and injured her pride in her ability to independently manage her complex physical care needs.” [36] | Fear of suffering and of the loss of control |
| “The patient said, ‘I don’t want strangers in my house. I’m doing fine. My wife’s taking care of me. I just don’t want people there 24 hours a day telling me what to do. And so I have had people refuse hospice because their understanding is that hospice takes control of their personal lives. They are very afraid of people coming in and they don’t want anybody to take over the role of their caretaker.’” [36] | Desire for independence  Importance of privacy |
| “I will do things my way and the hell with everything and everybody else. Nobody is going to talk me in or out of a darn thing… What will be will be; but what will be, will be done my way. I will always be in control.” [24] | Decision making |
| “One family member described her mother as an extraordinarily independent person, absolutely needing to be in control of her life all the time and already felt—how shall I put it—she had problems with feeling not in control.” [24] | Desire for independence |
| Participant: “I myself want to be in control as long as I can, I don’t want doctors and nurses controlling me [...] I’m on morphine, I get a lot of breakthrough pain, when I get to the pitch where I really can’t cope with anything any more, where my quality of life is totally gone, I will tell my husband I want a really good day out with the kids which is when he’ll know that when I go to bed that night I won’t wake up the next morning: [...] I think that you have to really look into it seriously, whether this is the right thing for the right person because I think there is the risk it might be abused. But with myself, if the legislation was there then it would be nicer for me, so I’m not on my own, which I know I will be because I don’t want any of the family here when it happens.” Interviewer: “Why don’t you want anyone with you?” Participant: “Because I don’t want them involved, I don’t want them to get in trouble. [...] In other countries, [...] I believe now, you’re allowed to choose when you die so you’ve still got your dignity. This country we don’t allow it; [...] if anybody helps us they lock them up, which is wrong. You’ve taken away that person’s dignity and nobody should have the right to do that. We should all have the right to choose when we die and how we die”. [42] | Loss of the value of life Desire for self-determination |
| “I never thought about giving up but my fear was that I didn’t know much about cancer. There are so many people that linger, and I was afraid that I could not cope. I know I will die, but I don’t want to be lingering and suffering and people around me to suffer with me. So I thought, ‘I will go for a swim’ and I don’t know how to swim or I would go to a place like Holland. I just don’t want to be lingering, like people that can hardly talk and are really suffering and I don’t want to do that. I t’s the only thing that makes me feel a little bit emotional. I don’t want to deal with it so I think I would speed up things myself. I don’t want to be lingering here in palliative care, lying day and day, slowly dying. Oh no, I don’t want to do that. So, suicide is a way of exiting. I don’t want to talk about that because I like life and I have lots to live for, but if I come to the point when I am too weak to do anything, then I don’t want to stay.” [39] | Fear of suffering and of making others suffer Desire to hasten death |
| **Subtheme: Desire for self-determination** | |
| “So she was a control person. You know, we are talking big time control... You know, I am in charge here. She sort of self-directed her medical care... It was a control issue, not a pain issue...’I want to be in control of my destiny. I don’t want to go out as, you know, incontinent, in pain, crying, you know tearful person. I want to go out with some dignity’”. [34] | Loss of functionality Loss of the value of life Desire for self-determination |
| “She just felt this was not dignified at all for a woman who had been in control all of her life. And she knew the end was near anyway. And she said, ‘I want to do it on my terms. I want to choose the place and time. I want my friends to be there. And I don’t want to linger and dwindle and rot in front of myself.’” [34] | Desire for control over the end-of-life process Desire for self-determination |
| “When I saw her she was very, very weak and very dehydrated. And again, I told her, I said, ‘Gee, you’re within a couple days probably of losing consciousness just from dehydration, and we could make sure that you just slept and did not suffer and it would just be a short time.’ She had the 15-day wait and she had 4 days before the medicine could be prescribed. And I told her that I didn’t think she would be able to do that unless she could solve the nausea and dehydration that she would last for 4 days consciously and to take the medicine. And she sort of struggled into a sitting position, asked her husband to get her a glass of water, and said, ‘I’ll get the fluids down somehow.’ And sort of forced… See, this is the paradox, this is where you learn that lesson about the control issue—she actually reversed the natural process to prolong her suffering, in order to be in control, to push the button herself.” [34] | Desire for control over the dying process Desire for self-determination |
| “I don’t want to undergo that (explicative) feeling of helplessness, that there’s not a (explicative) thing that I or anyone else can do, or “Sometimes I start yelling at my shrink that this is horrible, that why don’t I die right now? Why do I have to live through this? Related with: I don’t want to go through the dying process so I’ll kill myself.” [35] | Feeling of being useless Suffering Desire to hasten death Desire for self-determination |
| “I think that anybody with a terminal illness should have the right to assisted suicide. I think that should be a fundamental right people have. I wouldn’t like to be like that sick poor man who was in the news recently who put a plastic bag on his head. I mean, he must have felt really bad to be able to do that, and I’m sorry that he had to do that because he should have had a more dignified option, and he shouldn’t have had to do it by himself.” [39] | Desire for self-determination Right to choose |
